# Characterization of varietal effects on the acidity and pH of grape berries for selection of varieties better adapted to climate change

**DOI:** 10.3389/fpls.2024.1439114

**Published:** 2024-10-10

**Authors:** Marc Plantevin, Yoann Merpault, Julien Lecourt, Agnès Destrac-Irvine, Lucile Dijsktra, Cornelis van Leeuwen

**Affiliations:** ^1^ EGFV, Univ. Bordeaux, Bordeaux Sciences Agro, INRAE, ISVV, Villenave d’Ornon, France; ^2^ Château La Tour Carnet, Saint-Laurent-Médoc, France; ^3^ Pôle Scientifique, Bernard Margez Grands Vignobles, Bordeaux, France

**Keywords:** grapevine, climate change, malic acid, tartaric acid, inorganic cations, varietal traits, pH, *Vitis Vinifera*

## Abstract

Climate change is drastically modifying berry composition and wine quality across the world. Most wine regions with a history of winemaking are suffering from a loss of typicity and terroir expression because of climate change impact on berry components at harvest, including wine acidity, with total acidity decreasing and pH increasing. Such changes can have a major impact on wine stability and quality. One important option for adaptation is the selection of grapevine varieties better adapted to warmer and drier conditions. Weekly measurement of tartaric acid, malic acid, pH and titratable acidity from veraison until maturity were carried out on 51 varieties over seven years in two experimental plots. Varietal differences were shown for the rate of malic acid degradation during the ripening period, with some varieties metabolizing malic acid faster per unit of thermal time than others. Some varietal differences were also noticed regarding tartaric acid modulation, which can occur under exceptionally high temperatures. Differences in the dynamics of pH evolution in grape must over the growing season were evaluated and varieties characterized with regard to organic acids (tartaric acid and malic acid), inorganic compounds (cations) as well as pH levels and stability. This multi-trait approach allows the selection of grapevine varieties based on parameters linked to their acidity, which is of particular importance in the context of climate change.

## 1 Introduction

Grape berry juice contains a complex mix of acidic compounds that evolve over the growing season. The two main organic acids, which account for up to 90% of the total organic acids in berry juice, are dihydroxybutanedioc acid and hydroxybutanedioic acid, which will be referred to respectively by their common names: tartaric acid and malic acid (Carbonneau et al., 2015). While many other organic acids can be found in berry juice, including citric acid, succinic acid, pyruvic acid, ascorbic acid, coumaric acid, they only account for a small fraction of the acids in the berry juice and are usually neglected (Kliewer, 1966). One minor organic acid of interest is gluconic acid (a sugar acid) which is used as an indicator of *Botrytis cinerea* infection (Cinquanta et al., 2015). This, however, does not directly impact berry juice acidity, but rather the final stability of the wine (Barbe et al., 2002). Even though total acids account for less that 0.7% of the components of the berry juice (Boulton et al., 1999) they play a major role in wine stability and sensory characteristics of wine.

Most plant species accumulate malic and citric acid, but high tartaric acid accumulation is unique to the members of the Vitaceae family and especially *V. Vinifera* (DeBolt et al., 2006). This relationship is so unique that presence of tartaric salts on ancient vessels is used as an evidence of wine production in archaeology (Garnier and Maria Valamoti, 2016).

Tartaric acid is a di-carboxylic organic acid and is almost only found under its L(+) configuration in grape berries (Moreno and Peinado, 2012). It has two constants of dissociation, pK_a1_ of 3.01 and pK_a2_ of 4.05 (Ribéreau-Gayon et al., 2012). Biosynthesis of tartaric acid in grape berries peaks during the herbaceous phase of vine growth, reaching a maximum value at veraison (Conde et al., 2007; Carbonneau et al., 2015; Burbidge et al., 2021). This biosynthesis has long been under investigation and it has been shown that tartaric acid is not derived from oxidative metabolism of sugar (as with citric and malic acid), but rather results from a degradation of ascorbic acid (Loewus and Stafford, 1958; Saito and Kasai, 1969). This biosynthesis was not fully elucidated for plants until 2006 (DeBolt et al., 2006). Most of tartaric acid is biosynthesized within the berry itself, rather than transported from the leaves (Ruffner, 1982a, b; Hunter and Ruffner, 2001).

Tartaric acid content remains stable after veraison until berry maturity is reached (Bigard et al., 2019; Duchêne et al., 2014; Jackson, 2008), although its concentration decreases by dilution as berry volume increases (Bigard et al., 2019). While stable tartaric acid content (when expressed in mass per berry) during ripening is largely accepted in the literature, some older references reported breakdown of tartaric acid through respiration during this period (Peynaud, 1947; Takimoto et al., 1976). Tartaric acid content can also sometimes vary with potassium uptake due to water influx in the grape berry (Moreno and Peinado, 2012). Decrease of tartaric acid content is also reported under particular conditions, such as late harvest (Bondada et al., 2017), or grape berry drying (Rösti et al., 2018).

Tartaric acid is considered to be the most important acid in the final wine, with a tart-like taste (Volschenk et al., 2006) and a significant effect on wine astringency. Interestingly, the lower the tartaric acid content, the higher the astringency of the wine *(* Zhao et al., 2023). This counterintuitive relationship is due to the ability of tartaric acid to bind with proteins at low pH, which impacts the astringency more than the associated reduction in pH induced by higher levels of tartaric acid (Batista et al., 2010). Indeed, the negatively charged tartrates (due to the presence of anions), electrostatically react with positively-charged proteins, but also with different phenolic compounds such as highly-polymerized tannins and anthocyanins (Batista et al., 2010; Correa-Gorospe et al., 1991). Of additional importance, aside from its reactivity with monovalent cations, tartaric acid is otherwise very stable as it is not broken-down by microbiological activity (Jackson, 2008).

Malic acid is also a di-carboxylic organic acid. However, it is mostly found under its L(-) form in berry juice and differs from tartaric acid by having only one hydroxyl group in its chemical structure (Moreno and Peinado, 2012). As such, its pK_a_s are higher compared to tartaric acid (with pK_a1_ = 3.46 and pK_a2_ = 5.05; Batista et al., 2010). Malic acid is also known for having a lower acidifying power with less impact on the pH than tartaric acid (Gancel et al., 2022).

Malic acid accumulates in grape berries during the herbaceous development phase of the vine and reaches a maximum at veraison (Carbonneau et al., 2015). It is a major intermediate within the tricarboxylic acid (TCA) cycle and is thus formed from the glycolysis of sugars. It can also be synthesized from phosphoenolpyruvate (PEP) after CO_2_ fixation during photosynthesis (Carbonneau et al., 2015; Volschenk et al., 2006). In return, malic acid is easily decarboxylated to PEP, respired or consumed in the sugar gluconeogenesis, which explain the higher instability of malic acid compared to tartaric acid (Jackson, 2008).

Due to the lower availability of sucrose for providing energy through respiration at veraison (because of chlorophyll degradation), berries move their respiratory metabolism from sugar to malic acid, which leads to a decrease of malic acid during grape ripening (Volschenk et al., 2006; Keller, 2010; Shahood et al., 2020). This shift has sometimes been found to be slightly desynchronized, with malic acid degradation happening after the start of the increase of berry sugar, but only under cool conditions (Rienth et al., 2016). As such, malic acid is the major organic acid in the berry at veraison, with values up to 25 g/L, but quickly decreases in concentration in the berry to values at maturity ranging from 1 to 5g/L (Ribéreau-Gayon et al., 2012).

Interestingly, this decrease of malic acid content is not only a physiological characteristic, but is also driven by climatic conditions, with an increased rate of malic acid break-down at higher temperatures due to a higher enzymatic activity (Volschenk et al., 2006). In immature grape berries (during the herbaceous phase), a study reported that the greatest malic acid accumulation occurs between 20°C and 25°C, while higher temperatures tend to slow down the biosynthesis of malic acid (Lakso and Kliewer, 1975). This higher berry malic acid content in vines grown under cool climatic conditions has been repeatedly reported in the literature (Boulton et al., 1999; Volschenk et al., 2006; Carbonneau et al., 2015; Jackson, 2008; Rienth et al., 2016).

An increase in pH during maturity is also observed, partly explained by malic acid break-down, but also by the uptake of inorganic cations, especially potassium (K^+^), which neutralizes tartaric acid (Boulton et al., 1999). Potassium is accumulated in the berry pre and post-veraison and is the major cation in the berry at maturity (Carbonneau et al., 2015). It has a fundamental role for grapevine physiology, with importance in enzyme activation (Walker et al., 1998), cellular membrane transport (Walker et al., 1998), and osmotic potential regulation for controlling the plant water relations (Mpelasoka et al., 2003).

Potassium associates with free tartaric acid to form tartaric salts and, as such, decreases the tartaric/malic ratio and increases juice pH during berry maturation (Mpelasoka et al., 2003; Jackson, 2008; Rogiers et al., 2017). However, the relation between potassium and pH is relatively complex. Indeed, pH is influenced by several factors, including the amount of free and bound organic acids, their dissociation constants, the content of monovalent cations, and the titratable acidity (Ribéreau-Gayon et al., 2012). These parameters influence the buffering power of berry juice and wine, which is an effect of major importance in winemaking. Research by Peynaud (1947) found that the sum of cations, expressed as the sum of titratable protons (commonly referred to as total acidity) and the sum of mineral cations (commonly refered to as the alcalinity of ashes) is equal and thus in balance with the sum of anions, consisting mostly of malic and tartaric anions and a small proportion of phosphate anions. This balance of cations and anions was found to be constant from veraison to maturity in berry juice, despite the physiological changes occurring in the berry (Peynaud, 1947).

The buffering capacity of a solution such as grape juice or wine is associated with the ability of acids and their conjugate bases to reach a given equilibrium. This equilibrium can be presented as followed, with the exemple of an acid called HA:


$$\mathrm{HA}⇄{\text{H}}^{+}+{\text{A}}^{-}$$

If a strong acid is added to this solution, then the associated protons (H^+^) will bind with available anions (A^-^) in the solution to re-form the HA acid. For any addition of a strong base A^-^, a reaction with the free H^+^ in the solution will re-form HA. To the extent adequate protons or anions are available in the solution to bind with those being added, the pH of the solution will not change. This is referred to as the *buffering capacity* of the solution (Moreno and Peinado, 2012). In berry juice, partly salified organic acids are of greatest importance (Moreno and Peinado, 2012; Ribéreau-Gayon et al., 2012). Theoritically, the first acid to react as a buffer in grape juice or wine is tartaric acid, as it has the lowest pK_a1_, although, reactions with other acids are likely to be involved (Ribéreau-Gayon et al., 2012). This buffering capacity stabilizes the pH of grape juice and wine, and is an important factor in their organoleptic properties.

Climate change has been observed to impact these complex acido-basic balances in grape juice and in the finished wine. A sharp increase of pH due to higher temperatures has already been reported in many different wine regions across the world (Neethling et al., 2011; Barnuud et al., 2014; Adelsheim et al., 2016; van Leeuwen & Darriet, 2016; van Leeuwen et al., 2019). This increase of pH is mainly due to the direct effect of temperature on the rate of malic acid break-down (Fraga et al., 2012). However, evidence of increase of potassium with higher temperatures could also play a major role in the observed increase of wine pH (Mpelasoka et al., 2003; Mira de Orduña, 2010).

Many adaptations to such impacts of climate change on grape juice and wine composition are being investigated (van Leeuwen et al., 2024; Santos et al., 2020). One of those is the use of plant material better-adapted to future climatic conditions, including a change in grapevine varieties (Wolkovich et al., 2018). Although this way of adapting to climate change may be considered extreme, it is particularly powerful, as grapevine varieties show great phenotypic variability regarding traits such as phenology, berry composition, drought tolerance or heat tolerance (Destrac-Irvine et al., 2019; Delrot et al., 2020; Plantevin et al., 2022). Varieties better adapted to climate change in Australia were identified with regard to berry composition, in particular concerning compounds related to acidity (Clingeleffer and Davis, 2022). The variability in malic acid, tartaric acid and inorganic cation content has also been studied in cultivars breeded from a cross of two varieties (Duchêne et al., 2014).

The research presented here investigated varietal differences across a range of 51 genotypes, over seven vintages, and from two different sites within the Bordeaux wine production area. Varieties were discriminated by the dynamics of their different acid components (malic acid, tartaric acid, inorganic cations) during grape ripening and based on the sensitivity of their pH to changes in those acid components. Results provide insights in the varietal differences in terms of acidity, which allows identifying potential varieties with suitable traits for adaptation to a changing climate. However, those findings still need to be confirmed in other sites with different climatic conditions.

## 2 Materials and methods

Data for this study were collected from two experimental vineyards in the Bordeaux wine production region of France. The first one is referred to as “Château La Tour Carnet”, the second one is referred to as “VitAdapt”.

### 2.1 Château La Tour Carnet

#### 2.1.1 Vineyard setting

The experimental plot is located at the Château La Tour Carnet in Saint-Laurent Médoc, 33112, France (45.146796, -0.794222). The parcel includes 25 different red *Vitis Vinifera* varieties, planted in 2013 on the Gravesac rootstock. Each row contains one cultivar with 150 vines per row. The soil is made of sandy-gravel. The vineyard is dry-farmed with all varieties being double Guyot-pruned and trained with a vertical shoot positioning trellis at a density of 8,888 vines/hectare. Soil under the vines was tilled to destroy weeds and the inter-row was frequently mowed to limit excessive competition with the cover crop.

#### 2.1.2 Grape ripening monitoring

The evolution of major berry compounds was monitored weekly for the 25 varieties in 2022 and 2023. The analysis started a few days after mid-veraison which was assessed on twenty locations over each row for each variety using the methodology developed in Destrac-Irvine et al. (2019) (based on berry palpation) and continued until harvest for each variety. Harvest took place for every variety at optimum maturity for the intended wine style, assessed by standard enological parameters and berry tasting, and occurred generally between the end of August and mid-October. Harvest dates were also dependent on climatic hazards and the sanitary status of the considered variety, as in a classical production context. On the last sampling date (a few days before harvest) three analyses were implemented for each variety at three different sampling locations along the row and the values for each of the berry compounds were averaged for each variety.

Analyses were carried out on the juice extracted by gently pressing from 100 berries randomly selected along the row of each variety. Berries were sampled at different positions around the bunches to be most representative and collected in plastic boxes. Berry juice was analyzed for soluble sugars, total acidity, malic acid concentration, tartaric acid concentration, berry weight and pH by the Rolland laboratory (Pomerol, 33500, France). The laboratory uses a WineScan™ analyzer according to the method “Must” provided and calibrated by the manufacturer (FOSS, 92000 Nanterre, France).

### 2.2 VitAdapt

#### 2.2.1 Vineyard setting

The VitAdapt plot is located at Domaine de la Grande Ferrade, INRAE Nouvelle-Aquitaine Bordeaux Research Center in Villenave d’Ornon, 33140, France (44°47’23.8”N, °34’39.3”W). The parcel of 0.72 ha was planted in 2009 and includes 46 different *Vitis Vinifera* varieties grafted on the rootstock Selection Oppenheim number 4 (SO4). The design, including guard vines, represents 46 rows of 75 vines (row spacing 1.8 m; vine spacing 1.0 m; density 5,555 vines/ha). Five blocks were designed and sub-plots of 10 vines (spread over 2 rows) for each variety were randomly distributed over each block. The trunks are established 0.5 m above the soil surface, the vines are pruned as double guyot and hedged at 1.6 m. A cover crop is maintained every two rows (alternating every year) and weeds are eliminated under the row and in the row without cover crop by mechanical tillage. Diseases are controlled by integrated pest management practices.

#### 2.2.2 Grape ripening monitoring

Grape juice analyses were carried out for the 46 varieties on the different blocks since 2017. First analysis started around mid-veraison (assessed using the palpation methodology described in Destrac-Irvine et al., 2019) and was repeated once a week until harvest. Harvest dates were decided based upon standard enological parameters and berry tasting.

For each analysis, 60 berries were randomly selected from each replicate and each variety. Berries were picked at different positions around the bunches to be representative and collected in plastic bags (Lateral BagFilter - Interscience, France). In the laboratory the berry samples were counted and weighed to determine berry weight. The juice is then extracted by pressing the berries between two metal blades (Bagmixer 400W – Interscience, France) and then filtered (Lateral BagFilter - Interscience, France) before being centrifuged at 20°C for 10 minutes at 10,000 rpm. The recovered supernatant (minimum 12 mL) was then analyzed by Fourrier Transform InfraRed Spectroscopy (FTIR), using a WineScan™ analyzer according to the method “Must” provided and calibrated by the manufacturer (FOSS, 92000 Nanterre, France).

One large dataset was then created encompassing data from the two experimental vineyards. Varieties, replicates, vintages and locations are presented in **Supplementary Materials**, **Supplementary Table S1**.

### 2.3 Data analysis

#### 2.3.1 Data pre-treatments

Laboratory results for tartaric and malic acid were first expressed in *concentration* (g/L). Using the berry weight, the two variables were then converted to μmol/berry (referring to *content*) and meq/L (referring to *concentration*). Inorganic cation content was estimated following the equation developed by Boulton, 1980:


$$\begin{array}{c} \mathrm{Inorganic}\;\mathrm{cation}\;\mathrm{concentration}\;\\ =\mathrm{Malic}\;\mathrm{acid}\;+\mathrm{Tartaric}\;\mathrm{acid}-\;\mathrm{Titratable}\;\mathrm{acidity} \end{array}$$

Indeed, assuming that at pH = 7 the organic acids are under their salified forms, then the difference between the sum of malic acid and tartaric acid and titratable acidity (the three components of the equation being expressed in meq/L) represents the concentration of inorganic cations in the solution (Duchêne et al., 2014). This equation has the advantage of being easy to implement to estimate inorganic cation concentrations, although not entirely precise, as it relies on the hypothesis that organic acids are completely salified at pH 7. Indeed, in grape must 7-10% of the organic acids may not yet be salified at pH 7 (Dienes-Nagy and Lorenzini, 2012).

#### 2.3.2 Statistics software

Statistical analyses were run on R Studio version 2022.12.0 using “Dplyr” package for descriptive analysis, “Stats” package for hierarchical clustering analysis (HCA) and “FactoMineR” package for principal component analysis (PCA).

## 3 Results

### 3.1 Varietal effect on components related to acidity in grape berries

#### 3.1.1 Tartaric acid modulation

**Figure 1:**
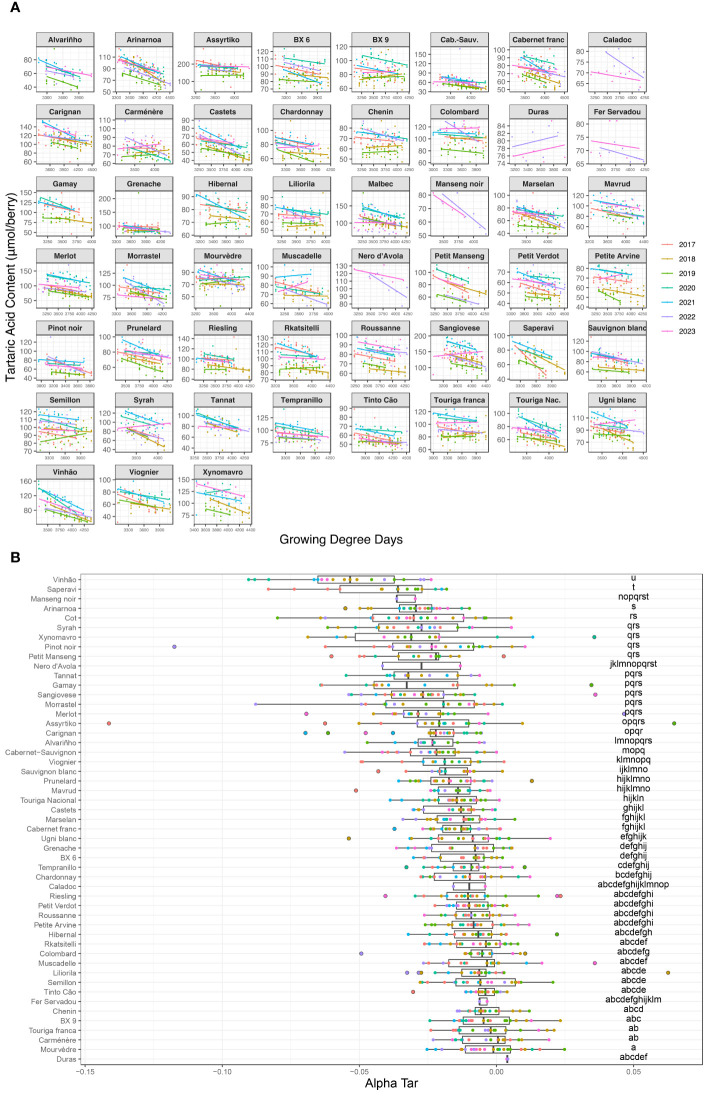
**(A)** Varietal differences in tartaric acid modulation, expressed as content (μmol/berry), from the 2nd week after veraison until the 8th week after veraison for the 51 varieties of the VitAdapt and La Tour Carnet experimental plots from years 2017 to 2023. **(B)** Varietal differences in tartaric acid decrease for the 51 varieties of the VitAdapt and La Tour Carnet experimental plots from years 2017 to 2023. The more negative alpha_tar, the higher the rate of decrease during grape ripening. Letters in the right column indicate statistically significant differences assessed with Tukey test. The black vertical line in each bow represents the median value. Colors represent the vintages, as displayed on the legend of panel **(A)**.

The evolution of tartaric acid content after veraison was investigated for the varieties of the VitAdapt and La Tour Carnet plot. Biases induced by machine calibration were identified on the first sampling date for tartaric acid estimation. For this reason, this evolution was assessed starting from the second week after veraison. Similarly, to avoid any degradation induced by late-harvest or berry drying, samples collected after the 8^th^ week after veraison were discarded. The kinetics of tartaric acid content were analysed as a function of thermal time (summation of mean daily temperatures starting from January 1^st^) in order to neutralize differences in phenology induced by varietal or year effect. The linear curves for each variety across all vintages are presented in **Figure 1A**.

**Figure 1B** presents the coefficients of the slopes (called “alpha tar”) for each variety, block and vintage shown in **Figure 1A** from 2017 through 2023. The more negative the coefficient, the higher the rate of decrease of tartaric acid for this variety, block and/or year. The level of significance of the differences were tested through an analysis of variance with Tukey significance classifications (at p<0.05). Most varieties of the study show a relatively stable tartaric acid content over the season, which is in line with the literature.

Two red varieties (Vinhão and Saperavi) are, however, significantly more sensitive to a higher decrease in tartaric acid content during the ripening period than the other varieties investigated. Malbec, Sangiovese, Syrah, Arinarnoa and Xynomavro also show negative coefficients and a decrease in tartaric acid content during the ripening period, although less pronounced.

#### 3.1.2 Malic acid degradation

The evolution of malic acid content from the first week after veraison until the 8^th^ week after veraison was investigated for the varieties of the VitAdapt and La Tour Carnet plot. Similar to tartaric acid content evolution, the rate of degradation was analyzed as a function of the thermal time (summation of daily mean temperatures starting from January 1^st^), in order to neutralize differences in phenology induced by varietal or year effect.

**Figure 2:**
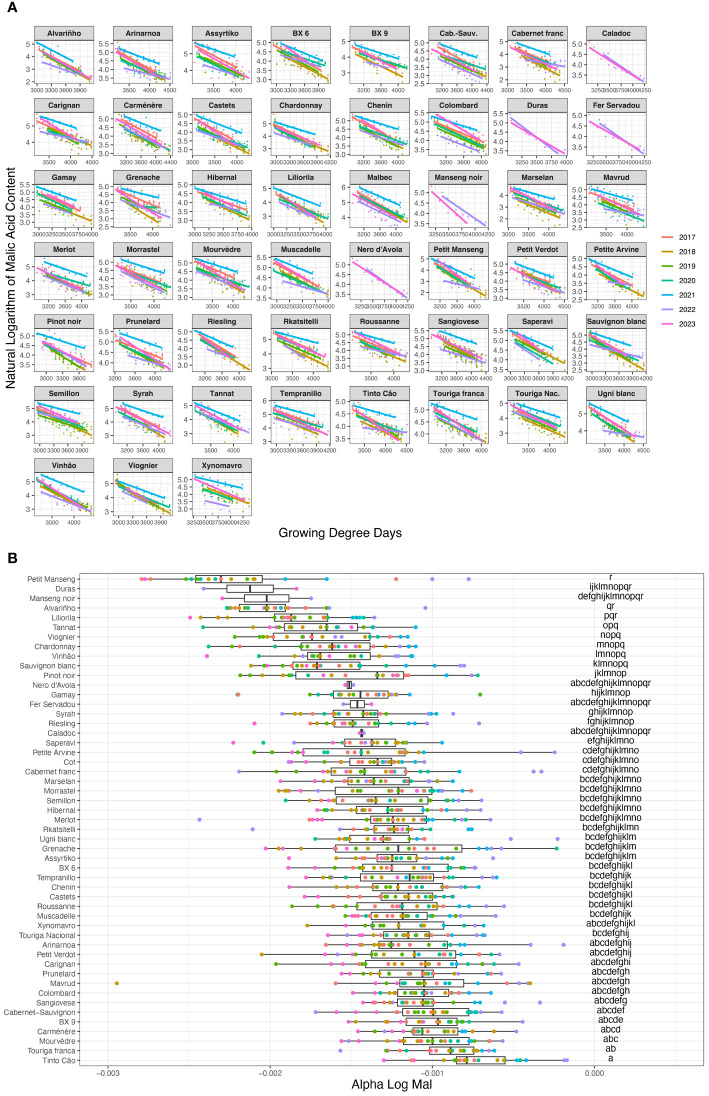
**(A)** Varietal differences in malic acid degradation for the 51 varieties of the VitAdapt and La Tour Carnet experimental plots from years 2017 to 2023 from the 1st week after veraison until the 8th week after veraison. **(B)** Varietal differences in malic acid degradation for the 51 varieties of the VitAdapt and La Tour Carnet experimental plots from years 2017 to 2023. The more negative alpha_log_mal, the higher the rate of degradation during grape ripening. Letters in the right column indicate statistically significant differences assessed with Tukey test. The black vertical line in each bow represents the median value. Colors represent the vintages, as displayed on the legend of panel **(A)**.

Malic acid content degradation follows a binomial curve with important degradation during the first four weeks after mid-veraison, followed by a rather slow degradation until maturity. To capture this trend, the natural logarithm of the curve of malic acid content was calculated and a linear regression of this logarithm as a function of thermal time was calculated from the 1^st^ week after veraison until the 8^th^ after veraison and is presented in **Figure 2B**.

**Figure 2B** presents the regression coefficients (called “alpha_log_mal”) of the 51 varieties for each block and each vintage. The more negative this coefficient, the more sensitive the variety is to malic acid degradation for a given heat summation. Vintage effect is clear, with cool vintages (such as 2021) presenting higher alpha_log_mal and hot vintages (such as 2019 and 2023) more negative alpha_log_mal. The alpha_log_mal was found to be highly correlated with the average temperatures from veraison until harvest, as well as with the average radiation over the same period (data not shown). Those results are in line with the literature (Lakso and Kliewer, 1975; Duchêne et al., 2014; Carbonneau et al., 2015; Rienth et al., 2016) and, as such, not presented in this study.

Varietal effect on malic acid degradation is substantial and forms a continuum from less sensitive to more sensitive. Varieties such as both Touriga (Nacional and Franca), Mourvèdre or Carménère tend to have a slow malic acid degradation compared to varieties such as Petit Manseng, Duras, Manseng noir or Alvarinho, which have a quicker malic acid degradation as a function of thermal time.

The possible effect of initial malic acid content at veraison (i.e., when the biosynthesis of malic acid has reached a maximum) on this alpha_log_mal was investigated as one could expect a higher rate of malic acid degradation when the initial malic acid content at veraison is higher (**Supplementary Materials, Supplementary Figure S3**). Except in 2022, no clear relationship was found between alpha_log_mal and malic acid content at veraison (**Supplementary Figure S3**). The coefficient alpha_log_mal, therefore, reflects the climatic and varietal effects on the rate of malic acid degradation and does not appear to be biased by the amount of malic acid at the beginning of this degradation period. Similar results were found when alpa_log_mal was plotted against the malic acid concentration at veraison (data not shown).

#### 3.1.3 Drivers of pH

##### 3.1.3.1 The evolution of the drivers of pH during the ripening period

###### 3.1.3.1.1 Varieties clustered

Mixed linear models were applied on the dataset to capture the main drivers of pH. The original dataset was divided week by week, starting at the 2^nd^ week after mid-veraison until the 8^th^ week after mid-veraison. The data from the first week after mid-veraison was discarded, because of a calibration error for tartaric acid. For each week, a linear model of the pH as a function of malic acid, tartaric acid and inorganic cations was created, with three random effects: variety, year and block. Then the three predictors of pH (tartaric acid, malic acid and inorganic cations) were scaled. Models were made with 396 points (for the 8^th^ week) to 1001 points (for the 4^th^ week). The marginal and conditional R^2^ can be found in **Supplementary Materials**, **Supplementary Figure S1**. The significance of the three predictors on the pH was tested by the mean of Type III analysis of variance (ANOVA). The three predictors had a significant impact on the pH at p-values<0.001 for all seven models (data not shown).

**Figure 3:**
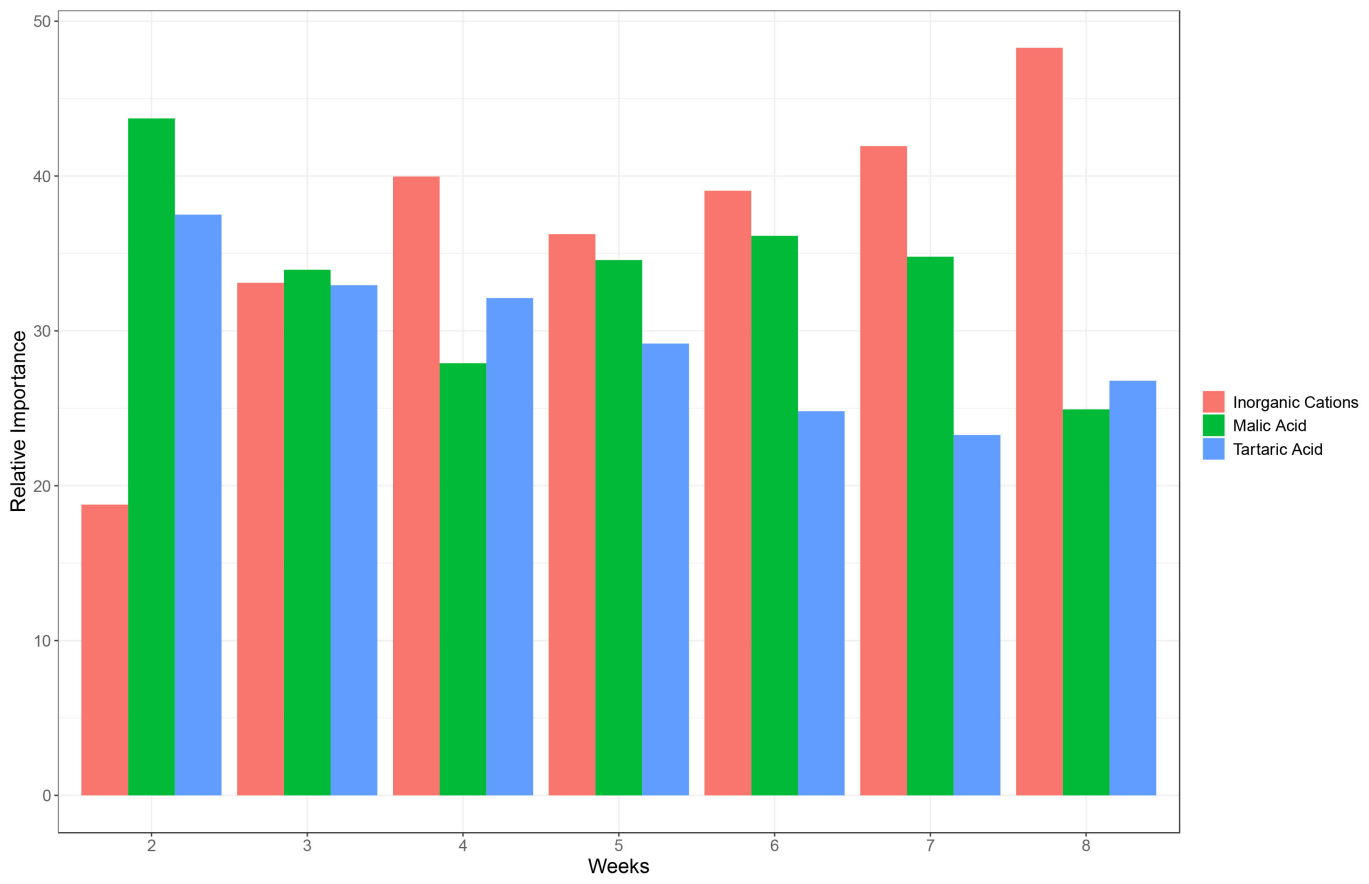
Relative contribution of the variance explained by inorganic cations, malic acid and tartaric acid of a mixed model assessing the drivers of grape must pH in weeks 2 to 8 after mid-veraison (all varieties considered together).

To account for the relative importance of each predictor on the pH, the absolute values of the contribution to the total variance of the model of the three coefficients (tartaric, malic, inorganic cations) were summed. Then, the proportion of each predictor on the sum values was calculated (**Figure 3**).

Tartaric acid plays an important role on the pH of grape must during the first weeks after mid-veraison, while its importance decreases later in the ripening period (**Figure 3**). The relative contribution to the pH of malic acid follows a similar pattern as tartaric acid with a very strong importance on weeks 2, 3, 5, 6 and 7 after mid-veraison (higher than tartaric). However, its relative importance sharply decreases in week 8. The relative contribution of inorganic cations to pH increases steadily from week 2 to 8 after mid-veraison and becomes the major driver of pH at the end of the ripening period.

###### 3.1.3.1.2 Varieties considered separately

The same modelling technique was performed variety by variety, to investigate whether the relative importance of the three pH drivers (tartaric acid, malic acid and inorganic cations) on grape must pH was variety dependent. Weekly models were created for each variety starting the 2^nd^ week after veraison until 8^th^ week after veraison. Only the varieties from the VitAdapt plot were considered as a considerable amount of data points is needed for each variety. This mixed linear model of pH as a function of tartaric acid, malic acid and inorganic cations included two random effects: block and year. The three predictors were scaled per variety for each model. Because of some missing data, not all the individuals (i.e., the varieties) had the same number of data points. Varieties with less than 6 data points in a given week were rejected. This explains why for some varieties the entire evolution of the pH drivers is not shown, but only results for 5, 6 or 7 weeks. The distribution of the marginal and the conditional R^2^ of the models per week can be found in **Supplementary Materials**, **Supplementary Figure S2**.

Interestingly, it appears that tartaric acid has a lower importance on the pH at the beginning of the ripening period when models are made on a variety basis, compared to models based on all varieties clustered. Two white varieties are an exception on this trend: Petit Manseng and Riesling.

**Figure 4:**
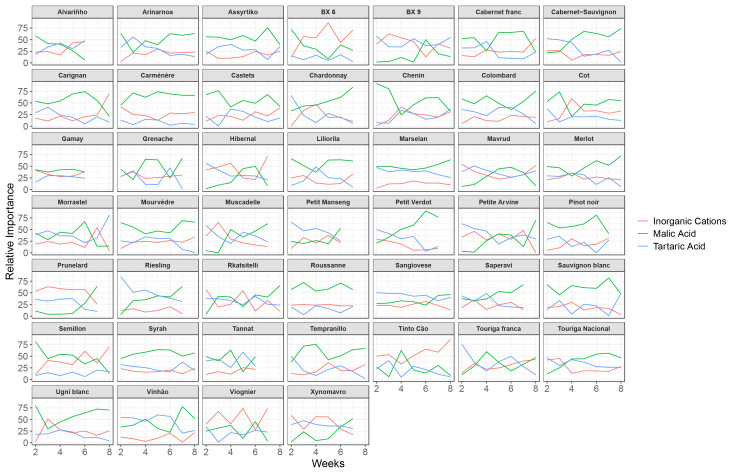
Relative contribution of the variance explained by inorganic cations, malic acid and tartaric acid of a mixed model assessing the drivers of grape must pH in weeks 2 to 8 after mid-veraison (each variety considered separately).

Tartaric acid is relatively stable during the ripening period for a given variety, which explains a limited effect of this metabolite on pH (**Figure 4**). Great differences in tartaric acid concentration do exist across varieties, which explains that when all varieties are considered together, tartaric acid becomes a major driver of pH. On the contrary, malic acid seems to have a stronger impact on pH (and very often the strongest of the three components considered in this study) when investigated on a varietal basis. Inorganic cations are an important driver of pH, both when considered on a varietal basis (**Figure 4**) or when considered for all varieties clustered together (**Figure 3**).

pH is clearly differently impacted by the three predictors for the different varieties, although some noise is introduced by changes over the season. Malic acid has a strong importance on pH variation during the entire ripening period for some varieties (Roussanne or Assyrtiko), for other varieties only at the end of the ripening period (Xynomavro or Petit Verdot), and for others only at the beginning of the ripening period (Alvarinho).

The pH of some varieties is strongly impacted by the inorganic cation accumulation at the end of the ripening period (Carignan, Chenin and Viognier), while the pH of other varieties is not as impacted by inorganic cation accumulation (Colombard, Syrah and Riesling).

### 3.2 Multi traits approach

For selecting varieties adapted to warmer and dryer conditions, it is important to have a global view of all components involved in grape berry acidity (alpha tar, alpha_log_mal and their impact on the pH). To achieve this objective, multivariate analyses were conducted. Generally, the pH of grape must increases during the first weeks of the ripening period to reach a plateau at the fifth or sixth week after mid-veraison (Terrier & Roumieu, 2001). Given these trends, the contribution of tartaric acid, malic acid and inorganic cations to the variance of pH were averaged for each variety at the plateau (i.e., from week 5 to week 8). It is important to identify varieties for which the pH is sensitive to changes in malic acid and/or inorganic cation concentration, because these components have a strong impact on the pH at the end of the ripening period when it is critical for the quality of the wine. The contribution of malic acid, tartaric acid and inorganic cations to the pH were averaged from week 5 to week 8 and included in the multivariate analysis together with alpha_log_mal, alpha_tar and average pH measured at week 5 for each variety.

**Figure 5:**
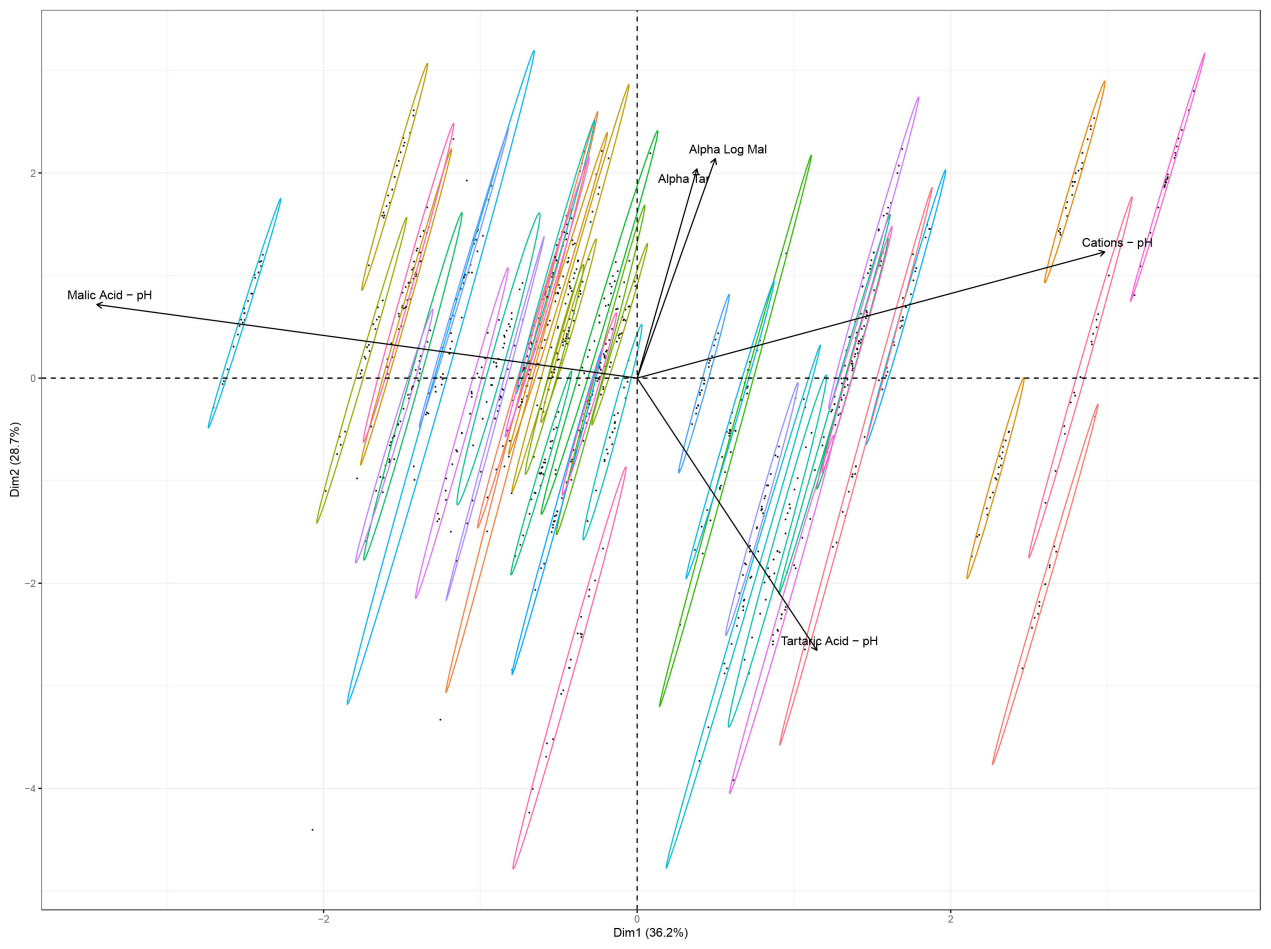
Principal Component Analysis of the alpha_log_mal, alpha_tar, malic acid impact on pH *(Malic Acid – pH)*, inorganic cations impact on pH *(Cations – pH)* and tartaric acid impact on pH *Tartaric Acid – pH)*. Ellipses are drawn around each variety barycenter (variety names were not added to the figure because it would harm clarity).

The selected variables were analyzed by means of a principal component analysis (PCA). Individuals (i.e., the varieties) and variables are plotted in **Figure 5**. More than 60% of the variance is explained with the two first dimensions of the PCA. The impact level of malic acid, tartaric acid and inorganic cations on pH sensitivity are all well represented on those dimensions. Alpha_tar and alpha_log_mal are less well represented on the first two dimensions of the PCA and thus less variability is explained by those variables.

Ellipses were drawn around the data points for each variety. Varieties are well separated, which is partly due to the fact that each variety has only one value for each predictors’ impact on pH. Hence, the drivers of the pH of grape must at the end of the ripening period are clearly variety specific and can be either inorganic cations, tartaric acid or malic acid.

**Figure 6:**
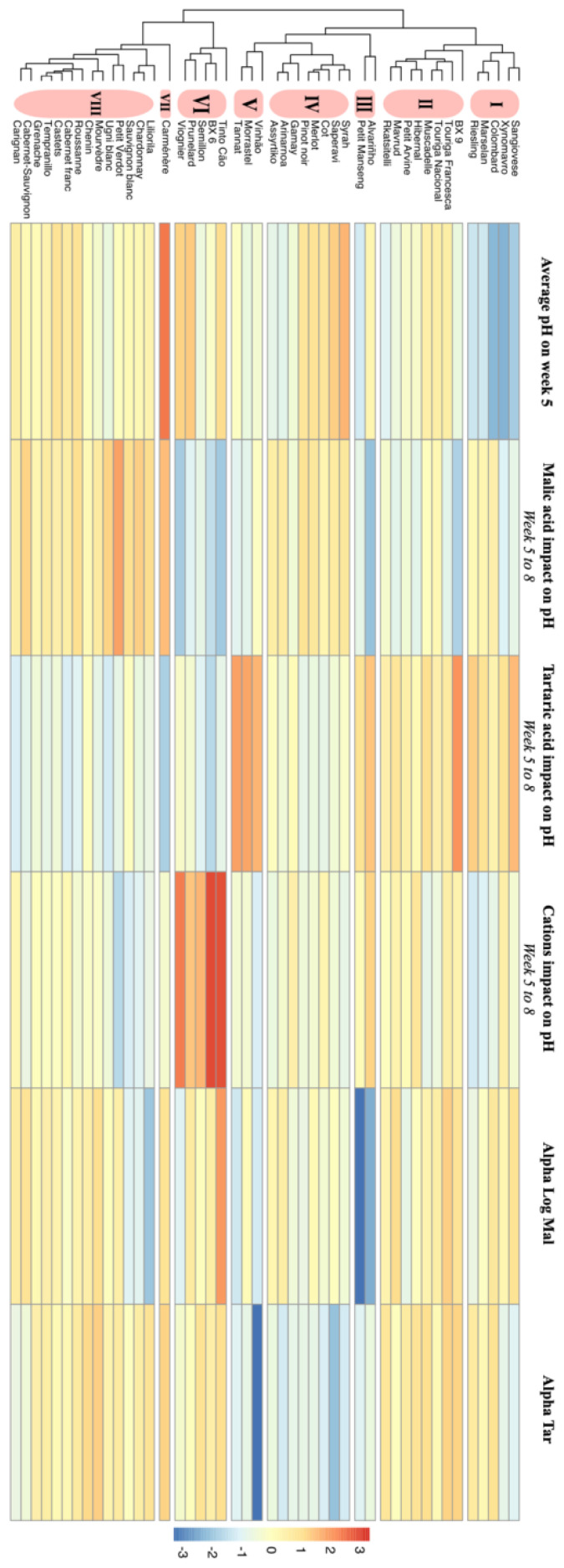
Hierarchical clustering analysis (HCA) of the 46 varieties from the VitAdapt plot based on average pH at five weeks after veraison, alpha_log_mal, alpha tar, inorganic cations impact on pH, malic acid impact on pH and tartaric acid impact on pH. Heatmap represents the scaled value for each variety on each component. HCA was implemented using the Ward method based on Euclidean calculations.

A hierarchical clustering analyses (HCA, using a Ward method based on Euclidean calculations) was performed on the varieties in order to group them according to their behavior in terms of pH and acidic components at the end of the ripening period (**Figure 6**). The following variables were included in this analysis: average pH on 5^th^ week after veraison (when it reaches the plateau), alpha_log_mal, alpha_tar and the three drivers’ impact on pH at the end of the ripening period. The heatmap of the HCA (based on Z-scores) represents the relative abundance of each of the variables for each variety (**Figure 6**). Eight clusters of varieties can be identified, with each cluster representing different behaviors with regard to their control over acidity in the grape must.

The first cluster includes the five varieties with the lowest pH of the study. The pH of these varieties tends to be sensitive to inorganic cations changes at the end of the season. Two of them (Sangiovese and Xynomavro) have a negative alpha_tar, meaning they are sensitive to tartaric acid degradation. Conversely, those varieties have a rather stable malic acid content.

The second cluster includes varieties with higher pHs compared to the first group (that could be considered as medium over the full range of varieties, except Rkatsitelli, which has a low pH). Varieties in this cluster are characterized by a low pH sensibility to malic acid changes but a high pH sensibility to tartaric acid at the end of the season. Varieties in this cluster do not seem particularly sensitive to malic acid or tartaric acid degradation (with the exception of the Petite Arvine, which has a more negative alpha_log_mal).

The third cluster includes two white varieties known for their high acidity: Alvarinho and Petit Manseng. Petit Manseng has a very low pH while Alvarinho is closer to the medium range of pH. These varieties share, however, a very negative alpha_log_mal (the two most negative) and as such tend to have a quicker malic acid degradation than other varieties. It is worth noticing that those two varieties have a rather low impact of malic acid on pH at the end of the season.

The fourth cluster is made of varieties with higher pH (except for Gamay, Arinarnoa and Assyrtiko). These eight varieties share one similar trait: a very low alpha_tar and as such are prone to tartaric acid degradation. As for the previous cluster, it is interesting to note that despite this low alpha tar, their pHs are poorly impacted by any degradation of tartaric acid, as evidenced by the low importance of tartaric acid on pH at the end of the season.

The fifth cluster includes only three red varieties which tend to have lower pHs. Those varieties are characterized with a high sensibility of pH to tartaric acid degradation with a rather negative alpha_tar and as such are prone to tartaric acid degradation (the lowest value is recorded for Vinhão).

The sixth cluster is composed of five varieties. They share an important common trait: inorganic cations have a very high influence on their pH at the end of the season (the highest influence over the entire panel of varieties) and, as a consequence, have pHs less influenced by tartaric and malic acid.

The seventh cluster contains only Carmenère, a minor red Bordeaux variety, more widely planted in South America. It has a very high pH and this was the reason for its separation from the other clusters. Its pH is more influenced by malic acid than tartaric acid or inorganic cations.

Finally, the eighth cluster encompasses 14 different varieties. Those varieties have an average pH and do not have any extreme behaviors. Their pH tends to be more influenced by malic acid than other acidic components. Three of those vareties tend to have a quick malic acid degradation as shown with their negative alpa_log_mal (Liliorila, Chardonnay and Sauvignon blanc).

## 4 Discussion

### 4.1 Tartaric and malic acid degradation

Even if tartaric acid content is generally considered as stable after veraison (Peynaud, 1947; Bigard et al., 2019), some researchers agree on the rather unstable status of tartaric acid under unusual winemaking conditions (Bondada et al., 2017; Rösti et al., 2018) or under extreme climatic conditions (Moreno and Peinado, 2012; Takimoto et al., 1976; Peynaud, 1947). Those climatic conditions are becoming more frequent in vineyards around the world due to global warming, which is why the issue of tartaric acid beak-down is gaining importance.

Our results clearly show that tartaric acid content often decreases during the ripening period, at different rates depending on the year (**Figure 1B**). This decrease of tartaric acid content is, however, rather small when compared to malic acid degradation, with average tartaric acid content loss over the ripening period for all the varieties varying from 30% (for the 2022 vintage), 25% (for the 2020 vintage), 27% for the 2021 vintage, 12% for the 2017 and 2018 vintages to almost 0% for the 2023 and 2019 vintages. This decrease of tartaric acid content is, however, not only a vintage effect but also a varietal effect (as shown in **Figure 1A** with significant different alpha tar across the varieties). To our knowledge, this is the first time a varietal effect on the rate of tartaric acid content decrease is shown. However, varieties cannot be classified as being either sensitive or not sensitive to tartaric acid decrease, there is in fact a continuum from varieties being more or less sensitive to this trait.

Malic acid degradation is an important feature of grape ripening and it is well known that malic acid degradation after veraison depends on climatic conditions, in particular temperature (Bigard et al., 2019; Duchêne et al., 2014; Keller, 2010; Rienth et al., 2016), with the rate of degradation depending on the variety (Duchêne et al., 2014). The concept of alpha_log_mal developed in part 3.1.2 of this article is an easy and straightforward way of quantifying malic acid degradation from mid-veraison until the end of maturity of a given variety in a given year. The fact that this alpha_log_mal does not depend on malic acid content at veraison (**Supplementary Figure S3**) is of major importance and reinforces the importance of this trait for varietal selection.

By using data from seven vintages, we were able to assess the varietal differences in malic acid degradation of the 51 studied varieties despite a strong vintage effect. **Figures 2B** shows that this varietal effect on malic acid degradation is also taking place on a continuum of sensitivity as a function of heat summation.

These findings are of major importance when considering the **Figures 5** and **6**. Indeed, even if malic acid content tends to be rather low at the end of the ripening period, the amount of malic acid content is still significantly impacting the pH of most varieties.

### 4.2 Influence of acidic components on pH: varietal and non-varietal effects

pH depends on many subtle acido-basic equilibriums within the berry juice. Those equilibriums are mostly depending on malic acid, tartaric acid and inorganic cations (Boulton, 1980) but other factors play a role on the pH, including the concentration of other organic acids, their dissociation constant, the concentration and nature of the amino-acids which may affect the buffering capacity of the berry juice or the interactions between the tartaric and malic acid, which is not yet fully understood (Dartiguenave et al., 2000a, b; Ribéreau-Gayon et al., 2012).

pH is of major importance in wine production and is one of the most considered values for supporting harvest and winemaking decisions (Jackson, 2008). Furthermore, pH has been shown to have a direct impact on wine taste, much more important than total acidity (Ribéreau-Gayon et al., 2012). As such, identifying varieties with a more stable pH are important in the context of climate change, which otherwise can cause a general increase in pH (van Leeuwen et al., 2024; Santos et al., 2020).

Our analyses first assessed the relative importance of tartaric acid, malic acid and inorganic cations on pH as a function of the number of weeks after the onset of the ripening period, with all varieties considered together (**Figure 3**). The adjusted R^2^ presented in **Supplementary Materials S1** clearly showed that those three acidic components have a considerable importance on the pH, as expected. The unexplained part is very limited and may depend on more complex chemical issues.

Analysis of factors driving pH shows that tartaric acid plays a major role in pH variation during the first weeks after veraison. The importance of tartaric acid is decreasing as the ripening progresses, while the influence of inorganic cations is increasing. The importance of these inorganic cations on pH seems to follow the same trend as inorganic cation accumulation in the berry during the ripening period (Carbonneau et al., 2015). Hence, it is likely that this importance is only increasing due to a higher content of inorganic cations in the berry.

Of major importance, the malic acid plays a significant role in increasing pH during the entire ripening period due to its natural degradation in the berry, and still has a major importance on pH at the end of the season (**Figure 3**), even if its levels are much lower compared to the beginning of the ripening period (Bigard et al., 2019). As a result, our findings on the varietal effect on malic acid degradation, expressed as alpha_log_mal (**Figure 2B**) is highly relevant for assessing the suitability of new varieties for changing climate. Malic acid is thermo-sensitive and as such varieties with less negative alpha_log_mal (such as Mourvèdre or Touriga Franca) are interesting in a climate change context, as they tend to maintain rather low pHs at harvest.

On a varietal basis, the influence of tartaric acid on pH is relatively small, as ranges of tartaric acid concentration are lower across varieties than when all varieties are clustered (**Figure 4**). Conversely, the importance of inorganic cations and malic acid on pH remains relevant on a varietal basis.

Berry composition is much affected by the variety due to differences in phenology (and hence differences in climatic conditions experienced during the ripening period, with early varieties being exposed to higher temperatures) and genetic differences in their metabolism (Sabir et al., 2010). These differences in berry composition clearly affect the pH and are drivers of pH regulation. To capture this equilibrium with multiple drivers, mixed linear modelling appeared to be an efficient tool for assessing the varietal differences in pH regulation. For instance, it is interesting to note that we did find vintage differences in the inorganic cation accumulation, but no varietal differences (data not shown). However, the importance of organic cations on the pH varies according to the variety (**Figure 4**).

In a context of climate change, it is necessary to identify varieties with a rather stable pH (such as Sangiovese, Riesling or Marselan), less exposed to any changes of inorganic cations and/or malic acid, which are both affected by either warmer conditions or heatwaves (Bigard et al., 2019; Villette et al., 2020). Our findings bring strong insights on the suitability of 46 varieties to warmer climatic conditions, in particular near the end of the ripening period (from the 5^th^ week after veraison, when the pH reaches a steady status). To capture these traits, the impact on pH of malic acid, tartaric acid and inorganic cations from the 5^th^ week to the 8^th^ week seems to be a major varietal characteristic, with largely differing behavior of the varieties studied with regard to their pH.

Vine water status was not taken into consideration in this study but is also reported to have an effect on acidic components and pH of grape must. Etchebarne et al. (2010) found that under irrigation berry potassium content was lower and berry total acidity higher compared to dry-farmed conditions, while no differences in malic and tartaric acid content were found, nor differences in pH. Malic acid (expressed in concentration) decreased with water deficit, while tartaric acid increased in Agrorgitiko grape berries in Nemea (Greece; Koundouras et al., 2006). No significant effect of vine water status was found on total acidity in this study. When grape ripening was expressed as the rate of increase in the sugar/total acidity ratio (S/TA), ripening speed varied with vine water status (van Leeuwen et al., 2023). The rate of increase in S/TA as a function of thermal time increased under moderate water deficit (down to -0.8Mpa pre-dawn leaf water potential) and decreased under severe water deficit (PDLWPs lower than -0.8MPa).

### 4.3 A multi-traits approach for selecting varieties better-adapted to climate change

The findings discussed in parts 4.1 and 4.2 need to be considered with the average pH of the varieties. Indeed, the traits discussed in this study (impact of inorganic cations, tartaric acid and malic acid on pH, alpha_log_mal and alpha tar) only refer to dynamic characteristics of the variety, which have to be considered in regard to the absolute value of the variety’s pH. **Figure 6** clusters varieties as a function of those dynamic traits and the average pH of the varieties on the 5^th^ week after mid-veraison (i.e., when the pH reaches a plateau).

Varietal selection for climate change adaptation is highly dependent on soils, micro-climate, economic viability, wine styles, wine markets and, as such, the characterization of a large pool of varieties is very important for viticulturists to make their plant material selection. Our approach discussed in part 3.2 (through the **Figures 5**, **6**) aimed at discriminating clusters of varieties with similar behaviors.

The clusters help to determine which varieties are the most suitable in a particular context. For instance, places where heatwaves are a major threat at the end of the season, such as in California or South Australia, viticulturists should consider planting varieties with a slower malic acid degradation, like varieties from the 6^th^ cluster. Interestingly, the varieties in this cluster (Tinto Cão or Semillon) are some varieties already planted in hot winegrowing regions (Douro Valley, Rhône Valley, Hunter Valley). However, the pH of those varieties are more sensitive to inorganic cations, which tend to increase with drought (Monder et al., 2021) and viticultural practices should then be adapted to limit cation influxes in the grape berries at the end of the ripening period.

In the places where the effect of climate change on wine pH is an issue, varieties from the 4^th^ cluster should be avoided. All of those varieties are characterized by a high rate of tartaric acid degradation under warmer temperatures and this trait can become an issue. It is interesting to notice that those varieties are globally planted in cooler wine regions around the world (Gamay in Beaujolais, France; Pinot noir in Burgundy, France; Cot and Merlot in Bordeaux, France). The only exception is the Assyrtiko which is grown in the warm region of Santorini (Greece). But this variety has a very low pH, hence this tartaric acid degradation may not be a major issue.

Another parameter to consider is the importance of inorganic cations on pH. Indeed, in regions where drought and heat are an issue (and where irrigation is either not permitted, or not possible because of limited fresh water resources), grapes tend to have a higher content of inorganic cations at the end of the ripening period (Villette et al., 2020). In these situations, varieties included in the 8^th^ cluster may be better adapted than varieties from the 6^th^ cluster. This cluster (number 8), containing 14 varieties, pH is less affected by inorganic cations and, as such, the pH at the end of the ripening period is not greatly impacted by inorganic cations variations. This interesting cluster can be sub-divided in two smaller clusters. On one side, some of those varieties have a more negative alpha_log_mal (expressing a high rate of malic acid degradation) while other varieties have a less negative alpha_log_mal. The three varieties with a more negative alpha_log_mal are white varieties which are traditionally grown in cooler climates (Sauvignon blanc, Chardonnay and Liliorila). The varieties in this cluster with higher values of alpha_log_mal (which also have low values of inorganic cations importance on the pH) are varieties traditionally grown in warm or hot climates: Carignan in Languedoc-Roussilon (France) Grenache in Southern Rhône valley (France), Aragon (Spain) or South Australia; Mourvèdre in Provence (France) or Jumilla (Spain, where it is called Monastrel); Cabernet-Sauvignon in Bordeaux (France) or Napa Valley (California); Roussanne in southern Rhône Valley (France).

In warm regions, varieties from the first cluster (especially Xinomavro, grown in Northern Greece or Sangiovese, grown in Tuscany, Italy) are of interest, as they have some of the lowest pH values. However, those varieties can quickly break down tartaric acid and as such should be avoided when heatwaves during the ripening period are frequent. Similarly, varieties from the 5^th^ cluster appear to be poorly adapted to warmer conditions, because of the combination of the following traits: a high influence of tartaric acid on pH and a high rate of tartaric acid degradation.

The six traits analyzed in this research regarding the evolution of the acidity during grape ripening (**Figure 6**) may be considered as positive or negative depending on the production context, and in particular the climatic conditions and the projected future changes in temperature. Our work provides insights on those varietal behaviors, allowing grape growers and viticulturists to easily assess the potential suitability of the varieties in their production context.

## 5 Conclusion

This work first aimed to characterize the evolution of the two main organic acids in the grape juice of 51 varieties, from veraison through maturity: tartaric acid and malic acid. By using seven years of replicated data, it was possible to assess the varietal sensitivity to malic acid and tartaric acid degradation. This varietal effect is rather small, taking place on a continuum from more or less sensitive. Considering that berry composition is changing from veraison to harvest, we hypothesized that the drivers of pH evolved during the berry ripening period. Tartaric acid, malic acid and inorganic cations affect the pH of grape juice in different ways, with a medium importance of tartaric acid at the beginning of the ripening period, while inorganic cations become more important at the end of this period. Malic acid has an important impact on pH during the entire ripening period. We then investigated whether the importance of these drivers of pH were variety specific. Our results clearly show that the pH of each variety is differently impacted by the three drivers considered. Finally, a multi-trait approach allows to characterize varieties as a function of six traits, including the final pH at the end of the ripening period. The characterization of the drivers of pH at the end of the ripening period provide support to viticulturists and winegrowers for choosing plant material adapted to their current and future climatic conditions.

## Data Availability

The raw data supporting the conclusions of this article will be made available by the authors, without undue reservation.
