# Supplementary material for: Characterization of varietal effects on the acidity and pH of grape berries for selection of varieties better adapted to climate change
Source: Front Plant Sci. 2024 Oct 10;15:1439114. doi: 10.3389/fpls.2024.1439114 (PMC11499634; doi:10.3389/fpls.2024.1439114)
Supplement: Supplementary file 1 [file DataSheet1.docx]

Figure S1 : R^2^ of the mixed linear models for all varieties clustered from week 2 to 8 after mid-veraison.

Figure S2 : Distribution of the R^2^ of the mixed linear models for each variety considered separately, from week 2 to 8 after mid-veraison.

Figure S3 : The rate of malic acid degradation, expressed as alpha_log_mal, as a function of berry malic acid content at mid-veraison, expressed in μmol/berry.


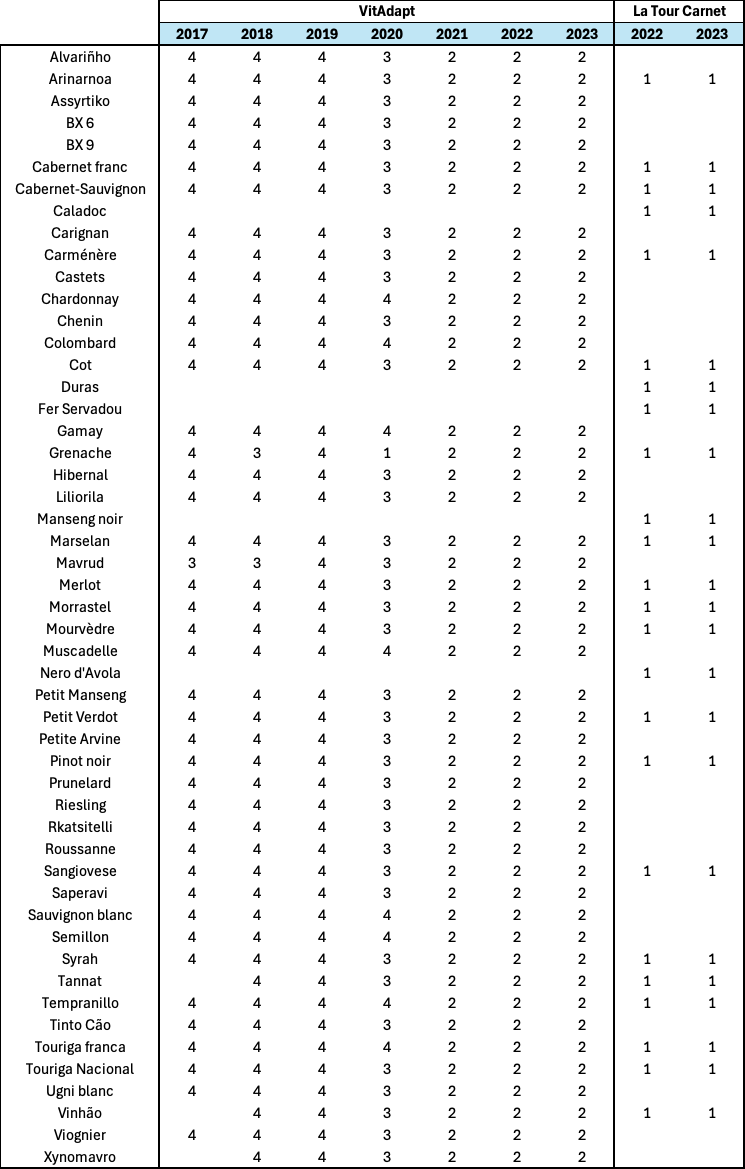


Table S1 : Varieties and their number of replicates for each vintage and each location.
